# Climatic niche shift in the amphitropical disjunct grass *Trichloris crinita*

**DOI:** 10.1371/journal.pone.0199811

**Published:** 2018-06-28

**Authors:** R. Emiliano Quiroga, Andrea C. Premoli, Roberto J. Fernández

**Affiliations:** 1 Estación Experimental Agropecuaria Catamarca, Instituto Nacional de Tecnología Agropecuaria (INTA), Sumalao, Valle Viejo, Catamarca, Argentina; 2 INIBIOMA, CONICET - Universidad Nacional del Comahue, Bariloche, Argentina; 3 IFEVA, CONICET - Cátedra de Ecología, Facultad de Agronomía, Universidad de Buenos Aires, Buenos Aires, Argentina; Universita degli Studi di Napoli Federico II, ITALY

## Abstract

Plant species disjunctions have attracted the interest of ecologists for decades. We investigated *Trichloris crinita*, a native C4 perennial grass with disjunct distribution between subtropical regions of North and South America, testing the hypothesis that the species has a similar realized climatic niche in both subcontinents. The climatic niche of *T*. *crinita* in North and South America was characterized and compared using presence records and five uncorrelated bioclimatic variables selected according to their ecological importance for the species. We used reciprocal modeling to make geographic projections of the realized niche within each subcontinent. Niche overlap between *T*. *crinita* distributions in North and South America was intermediate for the individual climatic variables and the multivariate space. In all cases the test of equivalence between climates inhabited by *T*. *crinita* indicated that the realized niche of the species differ significantly between subcontinents. Also, the similarity test showed that in the majority of cases the realized niche in both subcontinents was significantly different than that expected by chance. *T*. *crinita* occupied a greater diversity of environments in South than in North America, while in the latter its distribution was displaced to drier and warmer environments. The modeled geographic distribution using the actual occurrences of the species in North America did not accurately predict the distribution in South America, and vice versa. Together, these results led us to reject the hypothesis of similar niche of *T*. *crinita* in both subcontinents. This information may be useful to manage restoration efforts by presenting the suitable areas and climates for the species, and suggesting that translocation of individuals between subcontinents could only be recommended with caution because introduced genotypes can be potentially maladaptive, and could colonize sites actually not occupied by the species within each subcontinent.

## Introduction

Disjunct plant-species distributions have captured the interest of botanists and ecologists for decades [1, 2]. An interesting set of cases comprises several disjunctions of species and genera between North America and South America [3, 4], which recently have received a renewed attention ([5], and articles in that volume). On this regard, early studies comparing extant ecosystems of both American subcontinents in terms of their biotic and abiotic components (e.g. geomorphology, climate) have found high similarities [6]. Climatic factors are the main determinants of species distributions at broad scales (e.g. continental scale [7]). Climate influence on species distributions is a classic research topic in ecology, and still very active [8]. For organisms with disjunct distributions [9], as for invasive species, one of the first questions to be answered is whether the species inhabiting different regions occupy environments with similar climatic conditions [10].

Wiens [11] proposed that the niche concept enables the connection between biogeography and ecology. The niche concept was also suggested as a useful approach to study species inhabiting different regions [12, 13]. Hutchinson [14] defined the ‘niche’ as the set of environmental conditions in which a species can persist. He also made the distinction between the ‘fundamental niche’ and the ‘realized niche’ of a species, noting that while the former refers to the abiotic conditions, the latter also includes the biotic ones (e.g. interaction with other species by competition, facilitation, etc.) [15].

During the last 20 years, many techniques have been developed to characterize and compare species’ distribution and suitable environmental conditions [16]. Recent advancements in the field have been possible by the availability of species’ presence-data and environmental variables in publicly accessible online databases, and the development of specific analytical techniques, as species distribution models and multivariate analysis methods [17, 18]. Although these two groups of techniques are based on identifying environmental conditions of the sites occupied by the species (i.e. their realized niche -species in each site is influenced by biotic and abiotic conditions [7, 19, 20]), the former makes a geographic projection and defines areas with suitable environmental conditions for the species, while the latter aims to characterize and compare the niche in a multivariate space of environmental variables [16, 21].

The concept of niche conservatism refers to a taxon that retains similar ecological requirements across different geographical ranges or periods [11, 22]. Numerous studies have evaluated whether the niche of a species is conserved in geographically separated regions, and a debate took place around this topic [23, 24]. Almost all of these studies have been carried out on invasive species, considering their region of origin and the invaded one [10, 22, 25, 26]. However, testing this hypothesis during the invasion process has the weakness that the species probably has not reached yet the distribution equilibrium in the new region [10, 27]. Thus, the use of species that have naturally inhabited a region for a long time would be methodologically more appropriate.

Knowledge of the range of environmental conditions suitable for a species is important to understand its ecology and is relevant in conservation planning, e.g. for populations in marginal or extreme environments, and management strategies, e.g. restoration [28, 29, 30]. In this study, we investigate the realized niche of *Trichloris crinita* (Lag.) Parodi, a native C4 perennial grass disjunctly distributed in the subtropics of North and South America [31]. The species is promoted for use in restoration of degraded rangelands in both subcontinents [32–34]. Our objective was to compare the climatic envelope of the sites naturally occupied by *T*. *crinita* in North and South America, and to define suitable conditions for the species. The hypothesis is that the species has a similar, i.e. conserved, realized niche in North and South America. We tested two predictions related to this hypothesis:

i) In both subcontinents the species occupies environments with the same climatic conditions; and, ii) The geographic distribution modeled using presence records of *T*. *crinita* from North America will be consistent with the distribution of the species in South America, and vice-versa.

## Methods

### Study species

*Trichloris crinita* (Poaceae, Chloridoideae) is a warm-season, C4 perennial bunchgrass native of arid to subhumid rangelands of subtropical North and South America [31]. Previous studies have found that its distribution, similarly to that of other grasses, is strongly influenced by precipitation and temperature regimes [35, 36]. To date, there are no published studies about its phylogeography or its climatic niche in neither hemisphere. Preliminary phylogeographic analyses using nuclear and chloroplast DNA sequences showed a lack of between-subcontinents genetic structure (M. Paula Quiroga, pers.comm.). This suggests that, for at least the analyzed DNA regions, genetically alike *T*. *crinita* populations may have either diverged in recent evolutionary times from a common widespread ancestor or are the result of migration from north or south sources.

### Study area

Analyses were carried out in the American continent and nearby islands, including all South America and the portion of North America south of 49°N latitude. We excluded Alaska, Greenland and almost all of Canada, which have too cold climates to be occupied by the species. The boundary between Colombia and Panama was considered as a natural limit between the North and South subcontinents, and the Caribbean islands were considered as part of North America. In this way, the premise of considering the accessible area to the species on a relevant time scale was fulfilled (i.e. over its history, considering their dispersal ability [8, 37]).

### Species occurrence data

Coordinates of *T*. *crinita* occurrences were obtained from the open access online database ‘Global biodiversity information facility’ (GBIF, www.gbif.org; download date: 11/02/2013). A quality control was performed on the occurrence data [38]: a) eliminating repeated data; b) completing the coordinates of sites that had an accurate description of the site but lacked latitude-longitude information (using GEOLocate [www.museum.tulane.edu/geolocate] and Google Earth); and, c) eliminating data which coordinates did not match the description provided for the site. Finally, in order to achieve a spatial resolution similar to that of the climate data (see below) and to minimize possible effects of sampling bias and spatial autocorrelation [39, 40], coordinates closer than 2.5 sexagesimal minutes [~ 5km] were discarded (16 in North America and 9 in South America). This resulted in a presence data set of 104 and 177 coordinates for North and South America, respectively.

### Climate data

At the continental scale climate is the main determinant of species distributions [7]. Thus, a total of 19 biologically meaningful climatic (bioclimatic) variable layers corresponding to the study area were downloaded from the open access online database WorldClim (Version 1.4; www.worldclim.org). These variables were obtained by interpolation of climate data for the period 1950–2000 from meteorological stations located around the globe [41]. The spatial resolution of these data was 2.5 sexagesimal minutes (cell size = ~ 5km x ~ 5km). Of the entire set, five uncorrelated variables were selected by its biological importance for *T*. *crinita* [36, 42]. Correlation analyses were first performed between the 19 variables using ENMTools v1.4.4 [43] and those five with correlation coefficients of less than 0.85 were retained [19]. These five selected variables were related to the availability of water and temperature during the growing season and the entire year, as well as to the annual range of temperatures that the species will endure: annual mean temperature (AMT); temperature annual range (TAR, difference between the mean maximum temperature of warmest month and the mean minimum temperature of the coldest month); mean temperature of the warmest quarter (MTWQ); annual precipitation (AP); andprecipitation of the warmest quarter (PWQ).

### Realized niche characterization and comparison

To characterize and compare the niche of *T*. *crinita* in North and South America we carried out two types of analyses. In the first one, we considered climatic variables individually, while in the second one we used the five of them together in a multivariate analysis. Analyses were performed in R software, version 3.2.3 [44] using scripts provided by Broennimann et al. [17]. In both cases, we followed the methodology described by Broennimann et al. [17] and recommended by Guisan et al. [8], which consisted of three steps:

1. Calculation of the densities of species occurrences and available environments in North and South America, through axes determined by each climatic variable separately (first type of analysis), and in a multivariate space determined by the first 2 axes of a principal components analysis (second type of analysis). To do this, the axes were divided into cells, and the density values were ‘smoothed’ by a kernel density function [45]. Species occupancy of available environments were then calculated in a scale from 0 (no occupation) to 1 (maximum occupation).
2. Calculation of niche overlap, considering occupancy values from both subcontinents and correcting for differences in climate availability between regions. This was done by calculating Schoener’s D index [46] with values that range from 0 (no overlap) to 1 (complete overlap).
3. Statistical testing of ‘equivalence’ and ‘similarity’ between niches [47]. The equivalence test determines if two niches are equivalent or identical, by comparing observed D index [obtained in step (ii)] with a ‘null’ distribution of D values (n = 100; α = 0.05), each one of them calculated from two samples taken at random from a pool of all occurrences. The similarity test determines whether two niches are more similar than would be expected by chance (α = 0.05), by comparing the niche of the species in a region with randomly simulated niches in the other area. To do this, the observed D index is compared with two null distributions of D, which values are obtained by comparing simulated niches of a given region (n = 100, by random sampling of their environments) with the observed niche in the other region (i.e. simulated niches in North America vs. observed niche in South America; the comparison was then repeated in the opposite direction).

Additionally, using the smoothed occurrence of densities obtained in the multivariate space [step (i) above], we estimated the proportion of occurrences for each subcontinent that was in the overlap space between niches, and also those that were outside of the overlap zone and thus they represent unique occurrences to the niche within each subcontinent [10]. As the realized niche of a species may be limited in a region due to the absence of some climates (particular combinations of climatic variables), Guisan et al. [8] recommended to estimate these parameters considering all available climates in both regions, but also considering a range from the 100% to the 75% of climates common to both regions, to explore possible effects of ‘non-analogue’ and ‘less-frequent’ climates (the latter sometimes called ‘marginal’). Taking this into account, following [8], we made the estimates considering: a) all the climates of both subcontinents; b) all climates common to both subcontinents; c) climates common to both subcontinents after eliminating 12.5% of the less frequent climates; d) climates common to both subcontinents after eliminating 25% of the less frequent climates.

### Geographic projection of the realized niche

We applied the reciprocal modeling technique [48]: With the coordinates of occurrences of *T*. *crinita* in one subcontinent (either North or South America) we modeled the potential distribution of the species in that subcontinent, and the calibration was projected to model the potential distribution in the other. The modeling process was repeated 10 times in each direction, and results were averaged. We used the software MaxEnt 3.3.3k [49], which implements a machine-learning method to estimate the potential distribution of a species [49] and showed better performance for presence-only data than other species distribution modeling approaches [50, 51]. We used default settings, except that we used the option ‘fade by clamping’ to limit extrapolation to areas with climates outside the existent range in the calibration area [52].

Validation of the calibrated model for each subcontinent was performed by cross-validation (10 replicates) calculating the following metrics: the area under the receiver operating characteristic curve (AUC) [19, 50]; the difference between training data AUC and testing data AUC (AUCdiff) [53]; and the true skill statistic (TSS) [54, 55]. The AUC quantifies the model ability to discriminate coordinates of presence records respect to the rest of coordinates on the entire area (AUC = 1 indicates perfect prediction, AUC = 0.5 prediction expected by chance). The AUCdiff estimate the overfitting risk of a model (values closer to 0 indicates less risk). TSS compares the number of correct forecasts, minus those attributable to random guessing, to that of a hypothetical set of perfect forecasts (TSS = 1 indicates perfect prediction, TSS ≤ 0 prediction no better than random) [56]. In addition, we evaluated the importance of each climatic variable in terms of its contribution to the predictive power of the model in each subcontinent (using the jackknife option) adjusting models with each individual variable and also with each variable as missing (10 replicates), and then estimating the corresponding AUC values [49].

## Results

### Realized niche characterization and comparison

#### Individual climatic variables

**Fig 1:**
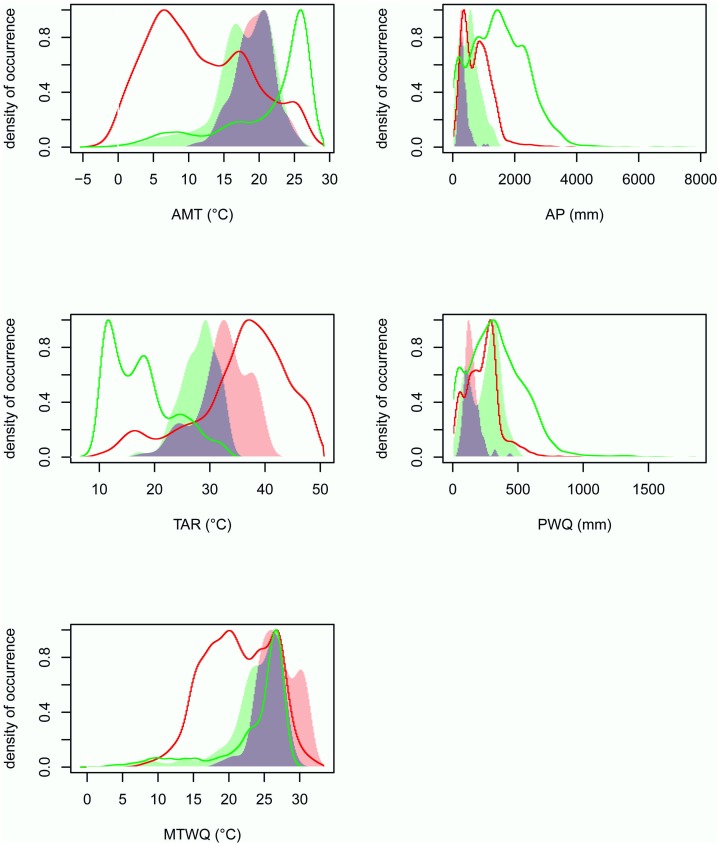
Densities of available climates (individual variables) and *T*. *crinita* occurrences in North and South America. Occurrence densities of *T*. *crinita* (in North America: red area; in South America: green area; overlap area between niches: violet) and available environments (in North America, red line; in South America, green line) for each individual climatic variable: annual mean temperature, AMT; temperature annual range, TAR; mean temperature of the warmest quarter, MTWQ; annual precipitation, AP; precipitation of the warmer quarter, PWQ.

Niche overlap between *T*. *crinita* distributions in North and South America, assessed via individual climatic variables, showed intermediate values (D = 0.4 to 0.7, Table 1, Fig 1). In all cases the equivalence test indicated that the niche of the species on both subcontinents is not identical (P <0.05 for all five variables, Table 1). On the other hand, the similarity test indicated that only for two variables in one direction of the test (TAR and MTWQ when comparing randomly simulated niches in South America to the realized niche in North America) the observed niche overlap was higher than expected by chance (P <0.05 in both cases). In the remaining eight cases (AMT, AP, PWQ in the same direction of the test, and all five tested variables in the opposite direction) no significant differences (P> 0.05) were found between observed niche overlap and that expected by chance (Table 1).

**Table 1:** Values of Schoener's D index and significance of equivalence and similarity tests between the niches of *T*. *crinita* in North and South America, considering each individual climatic variable and the multivariate space (last row).

Climatic variables	D	Equivalence	Similaritiy NAsim-SAobs	Similaritiy SAsim-NAobs
Annual mean temperature (AMT)	0.55	*	n.s.	n.s.
Temperature annual range (TAR)	0.70	*	n.s.	*
Mean temperature of the warmest quarter (MTWQ)	0.41	*	n.s.	*
Annual precipitation (AP)	0.56	*	n.s.	n.s.
Precipitation of the warmer quarter (PWQ)	0.49	*	n.s.	n.s.
Multivariate	0.35	*	n.s.	*

*, p<0.05; n.s., p>0.05. NAsim-SAobs: test considering simulated niches in North America and observed niche in South America. SAsim-NAobs: test considering simulated niches in South America and observed niche in North America).

In both subcontinents *T*. *crinita* occupied sites with annual mean temperature (AMT) lower than 27–28°C (Fig 1), but it was found more frequently in environments with AMT values between 15 and 22°C. The most notable difference between subcontinents was that in South America the species occurs in sites with AMT < 10°C, something that does not occur in North America, even though in this subcontinent there is a wide availability of environments with such temperatures, i.e. < 10°C. In both subcontinents *T*. *crinita* occupied sites with a temperature annual range (TAR) > 15°C, being the species more frequent in places with TAR near 30°C. The main difference for this variable between both distribution areas was that in North America a significant proportion of occurrences took place in sites with TAR between 35 and 42°C, while in South America no such climatic conditions are available (Fig 1). The species was more frequent in both subcontinents at sites with mean temperature of the warmest quarter (MTWQ) of 25–27°C (Fig 1). However, in North America it was also encountered in sites with MTWQ > 31°C, something that cannot occur in South America because environments with this characteristic were not available. On the other hand, the species occurred in sites with MTWQ < 17°C in South America, but not in North America despite of the availability of environments that meet this characteristic in the latter.

In both subcontinents the species was less frequent in sites with annual precipitation (AP) < 300 mm. In North America *T*. *crinita* commonly occupied sites with AP of 300–600 mm. Although it occurred even in sites with AP around 1000 mm, the species was not present in available, wetter environments. On the other hand, in South America the species occurred in sites with a wider AP range; while it was frequently found in sites with 400–1000 mm, it could also inhabit areas with AP near 1600 mm (Fig 1). With respect to the precipitation of the warmest quarter (PWQ), almost all occurrences of *T*. *crinita* in North America corresponded to sites with values < 300 mm (despite the existence of environments with higher precipitation), being more frequent in sites with PWQ between 100–200 mm. On the other hand, in South America the species occurred in sites with PWQ up to 500 mm, being more frequent in sites with 300–400 mm (Fig 1).

#### Multivariate analysis

**Fig 2:**
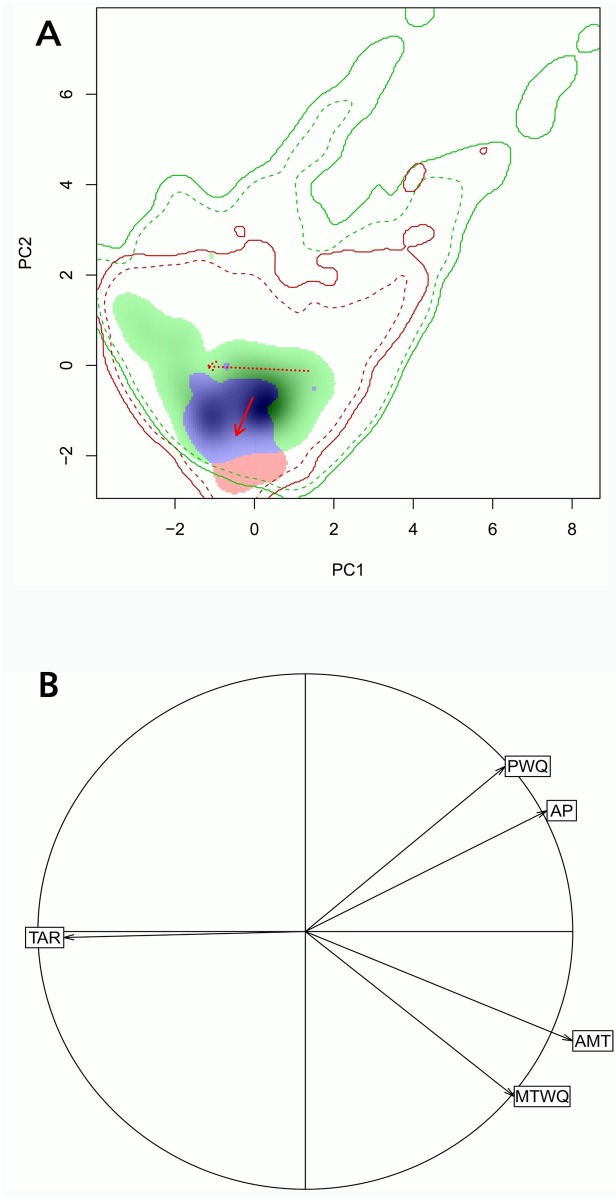
Densities of available climates (multivariate space) and *T*. *crinita* occurrences in North and South America. (A) Density of occurrences of *T*. *crinita* (shaded area, more dense shading indicates higher density) and available environments–climates-(continuous line = 100% percentile, dashed line = 75% percentile) in each subcontinent (North America: red; South America: green; overlap area between niches: violet) obtained through principal component analysis. Arrows (continuous line for occurrences, dashed line for environments availability) show centroid shifts in the South America to North America direction. (B) Correlation circle showing the contribution of climatic variables on each axis (annual mean temperature, AMT; temperature annual range, TAR; mean temperature of the warmest quarter, MTWQ; annual precipitation, AP; precipitation of the warmer quarter, PWQ). PC 1 and PC2 explained 64% and 20% of total data variability, respectively.

The first two axes of the principal component analysis explained 64% (PC1) and 20% (PC2) of the variability in data, respectively (Fig 2A and 2B). The PC1 was strongly and negatively related to the TAR, and was positively related to the remaining variables (Fig 2B). The PC2 was positively related to precipitation variables (AP, PWQ) and negatively to temperature (AMT, MTWQ). *Trichloris crinita* occurred in a greater diversity of environments in South America than in North America (Fig 2A). Its distribution in the northern subcontinent was displaced (and restricted) mainly to environments with lower precipitation (AP, PWQ) and to a lesser extent, to environments with higher temperature (AMT, MTWQ). Niche overlap between North and South American distributions of *T*. *crinita* was moderate (D = 0.35, Table 1, Fig 2A). Statistically significant differences were found between niches of both subcontinents (P <0.05 in the equivalence test, Table 1). However, the similarity test showed that the observed overlap was greater (P <0.05) than the expected if one compares the realized niche in North America with randomly simulated ones in South America. In the opposite direction, no significant differences were observed (P> 0.05; Table 1).

North and South America shared their climates in a large degree. However, the area corresponding to South America in the multivariate space was larger (i.e. it encompasses more diversity of climates), especially for having environments with higher precipitation not present in North America (Fig 2A). Except for a smaller proportion of occurrences in North America, the distribution of the species was almost entirely in climates shared by both subcontinents. This was evident when we observed the proportion of unique occurrences to each niche and those that were in the overlap area between North and South American niches (Fig 2A). These proportions kept almost unchanged when considering all available climates or the totality of shared climates between subcontinents (Table 2). However, the proportions of occurrences in exclusive climates and in the niche overlap space changed in North America (but not in South America) if 12.5 or 25% of the less-frequent climates were removed in each subcontinent. This indicated that a greater proportion of *T*. *crinita* occurrences in low-frequency climates existed in North America than in South America (Table 2).

**Table 2:** Proportion of *T*. *crinita* occurrences in each subcontinent corresponding to the overlap space between niches or that are exclusive to the niche of each subcontinent.

	North America		South America	
*Climate assumptions*	*Exclusive occurrences*	*Ocurrences in the overlap space*	*Exclusive occurrences*	*Ocurrences in the overlap space*
All the climates of both subcontinents	0.22	0.78	0.38	0.62
All common climates between both subcontinents	0.22	0.78	0.38	0.62
Common climates between subcontinents, after eliminating 12.5% of less frequent climates	0.06	0.94	0.37	0.63
Common climates between subcontinents, after eliminating 25% of less frequent climates	0.01	0.99	0.35	0.65

### Geographic projection of realized niche

#### Reciprocal models

**Fig 3:**
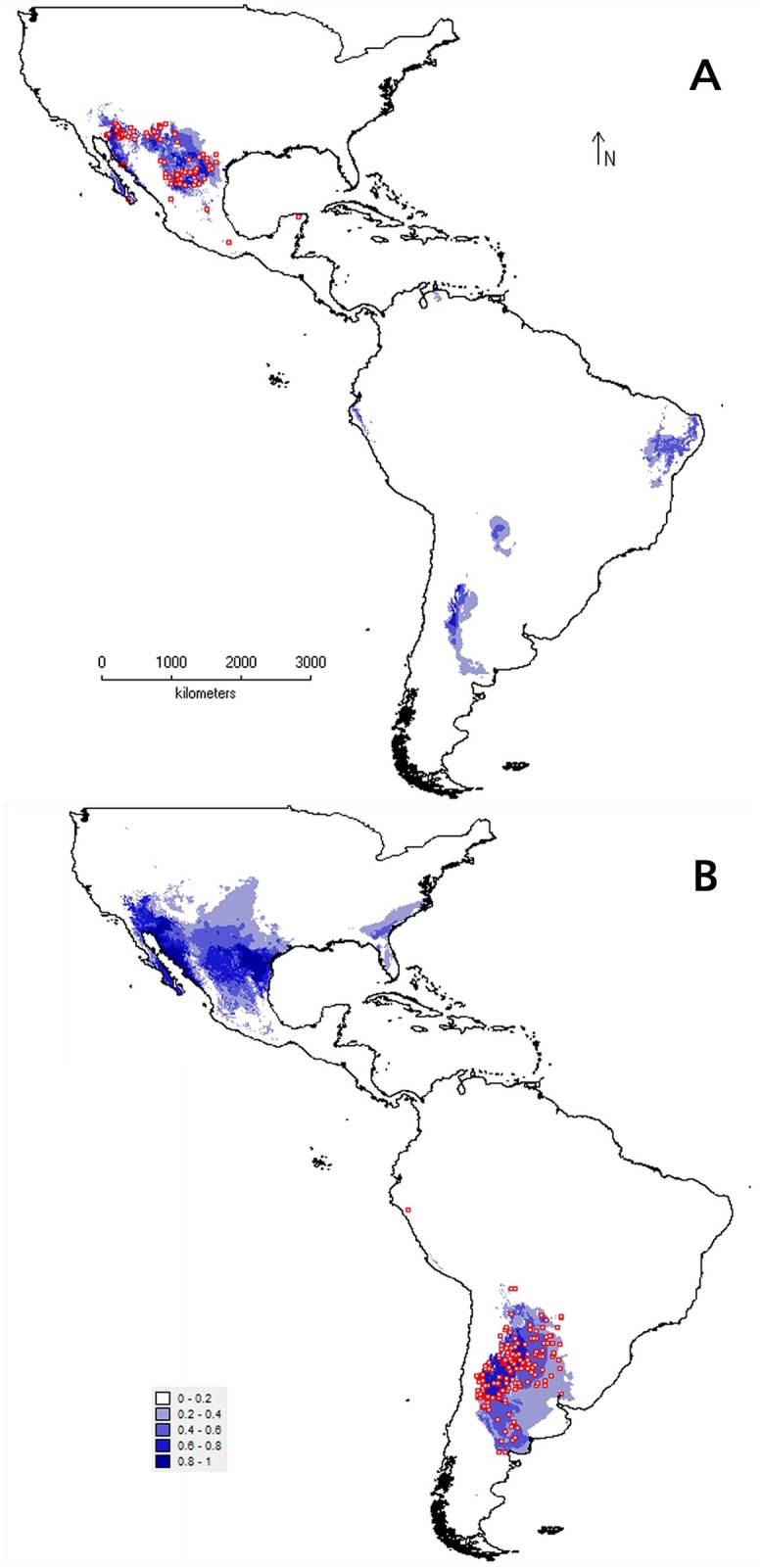
Geographic projection of the realized niche of *T*. *crinita* obtained by reciprocal modeling. (A) Model calibrated with North American coordinates (red squares) and projected to South America. (B) Model calibrated with coordinates of South America (red squares) and projected to North America. Suitability values range from 0 (areas with unsuitable climatic conditions) to 1 (areas with maximal climatic conditions suitability).

**Fig 4:**
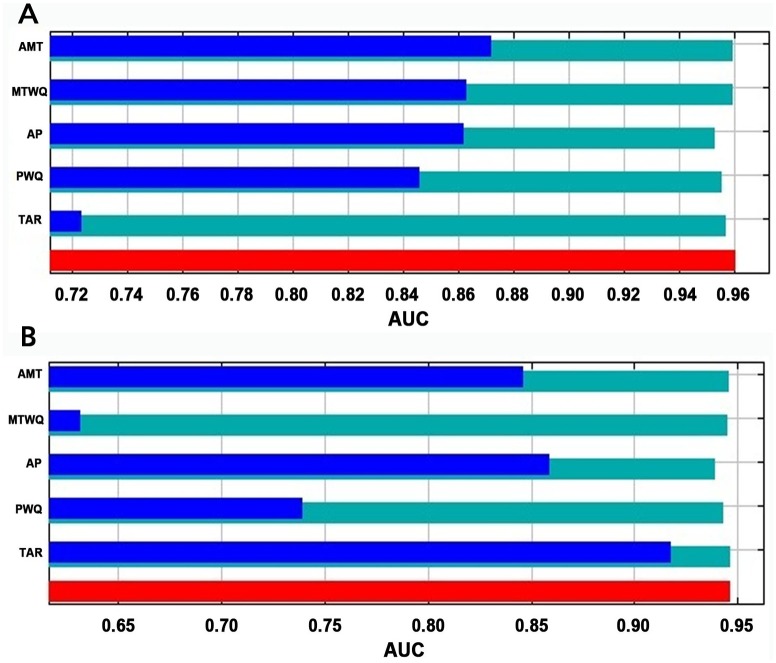
Predictive ability and climatic variables importance on the distribution models of *T*. *crinita*. AUC values (indicator of model predictive ability) obtained in the evaluation of the climatic variables importance on the distribution models of *T*. *crinita* in North America (A) and South America (B). Blue bars show AUC values obtained by using each variable individually. Cyan bars show AUC values obtained by excluding the focal variable and considering all the rest. Red bar shows the AUC value obtained by including all variables. Note: horizontal axes of both figures differ. Climatic variables are: annual mean temperature, AMT; temperature annual range, TAR; mean temperature of the warmest quarter, MTWQ; annual precipitation, AP; precipitation of the warmer quarter, PWQ.

Distribution models properly reflected the distribution of *T*. *crinita* in the subcontinent for which they were calibrated (Fig 3A and 3B). Models calibrated for North and South America showed high AUC (mean ± standard deviation: 0.96 ± 0.01 and 0.95 ± 0.02, respectively; Fig 4A and 4B) and TSS values (0.84 ± 0.07 and 0.80 ± 0.08), and low AUCdiff values (0.009 ± 0.013 and 0.011 ± 0.020). However, the projections of these calibrations from one subcontinent to the other were not equally accurate to predict the known occurrences of the species. Geographic projection on South America of the estimated niche in North America only predicted correctly the presence of the species in the northern (Bolivia, Paraguay, Peru) and southwest (Argentina) portions of the current range in South America. Potential habitats at the center and east of the actual distribution (Argentina) were not included. Also, areas of east Brazil and north Venezuela where no occurrences of the species are known, were predicted as potential habitat (Fig 3A). Thus, the projection on South America of the model calibrated in North America only coincides with 25% of occurrences (44 out of 177) and 21% of the range known for the species in the former subcontinent (0.35 x 10^6^ km^2^ of 1.64 x 10^6^ km^2^). In contrast, geographic projection of the South American niche to North America overestimated the distribution area in almost all directions, even indicating as potential habitat the peninsula of Florida, a region distant from the known distribution of *T*. *crinita* (Fig 3B). The projection correctly included 97% of the species occurrences in North America (101 out of 104, failing only 3 occurrences in southern Mexico), but indicated as potential habitat an area 164% higher than the known range of the species in that subcontinent (2.06 x 10^6^ km^2^ vs. 0.78 x 10^6^ km^2^).

#### Importance of climatic variables

The variable that individually showed the largest predictive power in the distribution model of *T*. *crinita* for North America was AMT (AUC = 0.87), although MTWQ (AUC = 0.86), AP (AUC = 0.86) and PWQ (AUC = 0.85) also showed high predictive abilities. On the other hand, TAR (AUC = 0.72) showed the lowest predictive power in this subcontinent (Fig 4A). In contrast, for South America, TAR was the variable that individually showed the highest predictive power (AUC = 0.92) in the distribution model (Fig 4B). Annual mean temperature (AMT, AUC = 0.85) and AP (AUC = 0.85), followed by PWQ (AUC = 0.74) presented gradually lower levels of predictive ability. However, in the southern subcontinent the lowest predictive power was achieved by MTWQ (AUC = 0.62). For each subcontinent, excluding the climatic variables ‘one-by-one’ almost did not diminish the predictive power of the model, obtaining in each case with the four remaining variables AUC values between 0.95–0.96 in North America, and between 0.94–0.95 in South America (Fig 4A and 4B).

## Discussion

*Trichloris crinita* showed a pattern of partial niche overlap between subcontinents (*sensu* Gallagher et al. [57], see below). We found that the realized niche of the species in North America is displaced to environments with somewhat lower precipitation (-200 mm of AP) and higher temperatures (+1.5°C of AMT) than in South America (Table 1, Figs 1 and 2). The shift may seem rather subtle, but is statistically significant; moreover, in our opinion it is biologically meaningful because it can translate into large water deficit differences. We also found that in South America the species covers a higher diversity of climates than in North America (Fig 2A). Then, our prediction that *T*. *crinita* occurs in both subcontinents in environments with the same climatic conditions was not fulfilled (Figs 1 and 2, Tables 1 and 2). Our results also contradict the prediction that the geographic distribution modeled using the occurrences of the species in North America would accurately predict the distribution in South America, and vice versa (Fig 3A and 3B). Together, these results led us to reject the hypothesis that the realized niche of the species does not differ between subcontinents and support the idea of a shift in the realized niche -defined as a change in the centroid or limits of the niche envelope in environmental space, *sensu* Guisan et al. [8]. Interestingly, our finding contrasts with previous results obtained in a study of 31 disjunct native (non-invasive) plant species of the European Alps and Scandinavia, where realized niche conservatism was the general pattern, and only one species showed a niche shift [58].

Studies of realized niche shifts in naturally disjunct species are scarce. Although those on invasive species are a particular case of extremely recent colonization, they showed mixed results respect to the occurrence of realized niche shifts between native and invaded areas [10, 57, 59]. However, as suggested by Petitpierre et al. [10] and Peña-Gomez et al. [27], niche comparisons between native and invaded regions share the aforementioned weakness: that invasive species probably have not reached the distribution equilibrium in the invaded area, thus increasing the probability of observing niche shifts. In contrast, the evidence of realized niche shift of a naturally disjunct species that we present here suggests that it is not advisable to assume *a priori* that the niche of a species would be conserved from a region to other (i.e. to predict areas of invasion) or from one moment to another (i.e. to predict future distributions under climate change).

The realized niche shift of a species may have a genetic component, because populations may modify their environmental tolerance due to differential selective pressures; genetic differences may also arise under restricted gene flow because of genetic drift and founder effects. Realized niche shift may also be caused by the interaction with biotic factors as natural enemies or symbionts. Both types of causes may be at work in *T*. *crinita*. Genetically based differences in adaptations to environmental factors were found between populations [60–62]. In addition, overgrazing by cattle may limit the distribution of the species [63] while the presence of certain arbuscular mycorrhizal fungi may favor it [64]. The realized niche shift that we detected would be consistent with an ongoing process of differentiation between northern and southern populations [65], but also with different influence of biotic interactions on both subcontinents [20].

Cavagnaro [35] and Cabido et al. [36] have pointed out the importance of temperature and precipitation for the distribution of several C3 and C4 grasses, including *T*. *crinita*, along altitudinal gradients of Argentina. The present study, performed at a wider spatial scale, further showed that the relative importance of climatic variables on *T*. *crinita* distribution varied between subcontinents (Fig 4A and 4B). Temperature annual range was the most important variable for the model adjusted in South America, but was the one with the least importance for the model adjusted in North America. This could be due to the predominance of tropical humid environments with relatively low annual temperature ranges in South America, in contrast to North America that consist of a larger continental mass at higher, i.e. cold, latitudes. So, the availability of suitable TAR conditions for *T*.*crinita* (between 20–40°C, Fig 1) could be a limiting factor for the distribution of the species in South America. In contrast, in North America, the distribution of the species seems to be more limited by the availability of warm environments (Fig 4A and 4B).

Our work depicted the range of climatic conditions that are suitable for *T*. *crinita* in North and South America. This information could be useful to manage restoration efforts: i) providing an estimate of the climates and suitable areas for the species, where it can be planted within each subcontinent, and ii) warning that transfer of germplasm between subcontinents should not be *a priori* recommended without further genetic research, because introduced genotypes can potentially colonize environments actually not occupied for the species in each subcontinent.
